# A repeated shuttle sprint test with female and male international field hockey players is reliable and associated with single sprint but not intermittent endurance performance

**DOI:** 10.1371/journal.pone.0271244

**Published:** 2022-07-13

**Authors:** Paul S. R. Goods, Alannah K. McKay, Brendyn Appleby, David Veli, Peter Peeling, Denise Jennings

**Affiliations:** 1 Murdoch Applied Sports Science Laboratory, Murdoch University, Perth, Western Australia, Australia; 2 Centre for Healthy Ageing, Health Futures Institute, Murdoch University, Perth, Western Australia, Australia; 3 Western Australian Institute of Sport, Mt Claremont, WA, Australia; 4 Mary MacKillop Institute for Health Research, Australian Catholic University, Melbourne, Australia; 5 Hockey Australia High Performance Program, Perth, WA, Australia; 6 School of Human Sciences (Exercise and Sport Science), The University of Western Australia, Crawley, Perth, WA, Australia; The Wingate College of Physical Education and Sports Sciences at the Wingate Institute, IL, ISRAEL

## Abstract

Field hockey is a high-intensity intermittent team sport that has recently undergone a series of rule changes that have resulted in a greater demand for repeated high-intensity movements. Coaches and practitioners now require a reliable assessment of repeated accelerations, decelerations and changes of direction to assess these important match qualities. This investigation assessed the test-retest reliability of a novel 6x40m repeated shuttle sprint test (20m + 20m with a 180° turn) and its association with 40m straight line sprint and YoYo Intermittent Recovery Test performance in 28 International field hockey players (n = 14 females and n = 14 males). The sum of 6 sprint times (SUM) demonstrated ‘excellent’ (ICC = 0.94 and CV = 0.59%) and ‘good’ (ICC = 0.84 and CV = 0.75%) reliability in females and males, respectively. Best sprint time during the repeated shuttle sprint test also demonstrated suitable reliability to evaluate field hockey physical performance (ICC = 0.92 & 0.76, CV = 0.76% & 1.00% in females and males, respectively). SUM was significantly associated with 40 m straight line sprint performance in females (r = 0.90; p<0.001) and males (r = 0.92; p<0.001), but only a weak association was found with YoYo Intermittent Recovery Test performance for either group (r = 0.20; p = 0.495 & r = -0.19; p = 0.525 in females and males, respectively). In summary, field hockey testing batteries that include a repeated shuttle sprint test should consider including a test of intermittent endurance. Further, changes in SUM greater than 1.0% can be confidently interpreted by coaches and practitioners as a real change for both female and male elite field hockey players.

## Introduction

Field hockey is a high-intensity intermittent team sport with a range of physical match demands including walking, running, sprinting, accelerations, decelerations, and changes of direction [1–10]. Relative to other team sports, field hockey is performed at a very high intensity (in excess of 130 m.min^-1^) [4]. In 2015, the rules of International field hockey were changed from two playing periods of 35 min, to four periods of 15 min. Subsequently, Sunderland & Edwards [8] have reported that field hockey players perform more high-speed running (10.8%) compared to earlier published time-motion analyses (5.6–6.2%) [11, 12]. Additionally, the authors reported that bouts of repeated sprints occur more frequently; however, direct comparisons with previous research are problematic due to differences in definitions or data collection methodologies employed [8].

Spencer et al. [11] published some of the earliest field hockey time-motion analyses which incorporated repeated sprint analysis, finding that 17 bouts of repeated sprints (minimum of 3 sprints, with a mean recovery time less than 21 s) occur during a match. Similar data was later reported by Lythe & Kilding [2] who found on average 37 high-intensity efforts were performed per game, with 16% of recovery times between efforts being <20 s, and a further 13% between 20–40 s. It has also been suggested recently that these earlier reports of repeated sprint activity in field hockey may have underestimated their frequency due to the use of velocity-based definitions only [9]. Recent team sport research has incorporated other high-intensity movements such as jumping or accelerating into investigations of repeated high-intensity bouts, which are associated with high energetic costs and warrant inclusion as a ‘high-intensity activity’ [10, 13]. One of the most recent time-motion analyses of elite field hockey has incorporated high-intensity accelerations and decelerations in addition to high-speed efforts [7]. While no data was reported on repeated high-intensity bouts, it was found that players performed 92–110 high-intensity acceleration and deceleration efforts throughout a match, which is higher than the 28 efforts previously reported under the old rules of 2 x 35 min playing periods [4]. Repeated bouts of acceleration have previously been found to occur more frequently than repeated-sprints in football referees [14], and when velocity-defined sprints and accelerations are considered together, bouts of repeated sprint activity in field hockey have been found to be more demanding and occur more frequently (3.6 bouts per match, compared to 0.5 for a velocity only definition) [9]. While these approaches to defining repeated sprints modified the durations, number of efforts per bout and recovery durations, they were still typified by short-duration, high-intensity efforts interspersed with brief recoveries, as per earlier velocity-based investigations [2, 4]. Taken together, this time-motion data clearly indicates that the frequency of repeated sprints in field hockey is increasing [8], that the incorporation of high-intensity movements with high energetic costs (such as accelerations, decelerations and changes of direction) into the definition of a sprint is warranted [9], and that when all high-intensity movements are considered, repeated sprint bouts occur more frequently than previously thought [9].

Repeated high-intensity movements have also been presented as critical for winning possession in team sports and reported to occasionally contribute to the outcome of a match [13, 15]. Therefore, it is vital that training interventions and testing procedures are targeted at these match-critical high-intensity running qualities. Accordingly, current testing protocols require updating to reflect International field hockey rule changes and subsequent changes in match running profiles. These newly designed tests will subsequently require validation in the elite populations who will undertake these assessment tasks. Investigations into field hockey testing procedures have shown that straight line 10 m & 40 m sprints [16], and hockey specific dribbling tests [16, 17], are able to discriminate between playing level. Additionally, a 6 x 30 m repeated sprint test developed for field hockey has been reported to be reliable in sub-elite athletes when results are presented as a sum of total sprint time [12], while 6 x 35 m repeated sprint test performance is associated with muscle-cross sectional area and low adiposity in national-level football players [18]. However, while traditional straight-line repeated sprint testing procedures incorporate an acceleration phase, they fail to include any change of direction or deceleration phase (which have been found to occur more frequently in most team sports) [19], and therefore, a repeated sprint test which incorporates these aspects is needed in field hockey. Tests which incorporate repeated accelerations, decelerations and changes of direction have been adopted by football [14, 20–22]; rugby [23], and other team sport athletes [24], where they have shown to be both valid and reliable [21]. These tests are associated with shuttle-based intermittent endurance tests [22], and elicit a greater physiological load than straight line repeated sprints [24]. Data on the reliability of repeated shuttle sprint performance and association with other physical performance testing data is currently lacking in field hockey. Without valid and reliable assessments of these physical qualities, coaches and practitioners are unable to quantify performance to inform training practices or to objectively assess athlete progression. Therefore, the aim of this investigation was to assess the reliability of a repeated shuttle sprint test in both female and male International field hockey players, and to explore the association of this data with performance in a straight-line, single sprint test and an intermittent, shuttle-based endurance test. It was hypothesized that a repeated shuttle sprint test would prove reliable in both female and male International field hockey players when assessed as a sum of total sprint time, and that this would be associated with single sprint but not intermittent endurance performance.

## Materials and methods

### Participants

A retrospective analysis of 2020 pre-season physical performance data was performed. The sample consisted of 28 elite field hockey players from the Australian National women’s and men’s squad (n = 14 females: 24 ± 3 y, 65 ± 8 kg, 169 ± 7 cm, 80 ± 12 mm sum of 7 skinfolds; and n = 14 males: 24 ± 3 y, 76 ± 8 kg, 179 ± 5 cm, 53 ± 15 mm sum of 7 skinfolds).

Written consent was obtained from each player via a signed data release form as part of the National Sporting Organisation’s athlete scholarship agreement. Institutional ethical approval to analyse this database was obtained prior to manuscript preparation (2021/ET000838).

### Experimental overview

Testing was conducted by National squad staff members (with assistance from the researchers in a service provision capacity), during one week in the general preparation phase of the pre-season period (December), across three consecutive testing days. Data was collected for athlete profiling purposes and to calculate test-retest reliability for use within the National Sporting Organisation. Subsequently, Institutional ethical approval was obtained to conduct retrospective analysis of reliability and associations with other physical performance measures for publication. The time of day and order of testing was kept consistent for all players, and included:

Day 1: Repeated shuttle sprint test, trial 1;

Day 2: Single sprint performance (three repetitions of 40 m separated by 5 min) followed by a 30 min rest and then the YoYo Intermittent Recovery Test, Level 1 (females) and Level 2 (males);

Day 3: Repeated shuttle sprint test, trial 2.

All tests were conducted following a standardized 15 min warm-up [23], consisting of 5 min light aerobic jogging followed by a dynamic stretching protocol and 2 submaximal sprints. A 3 min rest period then preceded the commencement of each testing session. All players were already familiar with the testing procedures prior to the data collection period. Players wore the same pair of running shoes for all tests, which was the same pair they would normally use for field testing, and any training not performed on the hockey field. Testing was conducted indoors in a temperature-controlled environment (21–25°C & 40–50% relative humidity). No other training was performed in the 48 h prior to, or throughout the testing period. Players were instructed to maintain their normal dietary habits throughout the testing week.

### Experimental procedures

#### Repeated shuttle sprint test

A repeated shuttle sprint test consisting of 6 repetitions of 40 m (20 m + 20 m with a 180° turn) on a 30 s time cycle was adapted from a previously validated protocol and conducted on a synthetic running track [20]. Players commenced the test from a stationary position at a line positioned 0.5 m behind a laser timing gate (Smartspeed Pro, Fusion Sport, Queensland, Australia) with all times recorded to the nearest 0.01 s. Players were instructed to sprint as fast as possible and then to place their foot on a line situated 20 m from the timing gate, before performing a 180° turn and sprinting 20 m back (all sprints were visually confirmed to have breached the turning line at 20 m or an invalid trial was recorded). Players decelerated to a stop at a cone placed 10 m behind the timing gate and then turned around and walked back to the starting line to await the next sprint. A verbal warning was given five seconds prior to the commencement of each sprint.

Three variables were derived from the repeated shuttle sprint data, including best single sprint time (usually the first sprint) (BEST), sum of 6 sprint times (SUM) and performance decrement (DEC) calculated as the percentage decrement from BEST (((DEC = SUM/BEST*6)*100)-100) as has been used previously [21].

#### Single sprint test

Players performed a 40 m straight-line sprint on the same synthetic running track as utilized for the repeated shuttle sprint test. Players commenced the test from a stationary position at the start line with laser timing gates positioned at 0 m, 10 m and 40 m (Smartspeed Pro, Fusion Sport, Queensland, Australia). All times were recorded to the nearest 0.01 s. Players were instructed to sprint the entire 40 m as fast as possible, with only the fastest 40 m trial from three attempts kept for analysis.

#### YoYo intermittent recovery test

Both the YoYo Intermittent Recovery Test Level 1 and Level 2 have been described previously and demonstrate good-to-excellent test-retest reliability [25, 26]. The Level 1 test is a measure of intermittent running performance with a high aerobic and anaerobic component towards the end of the test. While strongly correlated with the Level 1 Test, the Level 2 test elicits a higher rate of anaerobic energy turnover, and has been recommended for male team sport players [27]. In this investigation, the Level 1 test was used with female players, and the Level 2 test with male players as these are the tests which they regularly perform as part of their involvement with the Australian field hockey squads and were therefore familiar with. Both YoYo Intermittent Recovery Tests (Level 1 and 2) consist of 2 x 20 m shuttles which are performed at progressively increasing speeds, interspersed with a 10 s active recovery period, controlled by audio beeps. When a player twice failed to reach the finishing line before the next audio cue, the distance covered was recorded as the final test result. Both tests were conducted in the same indoor facility as the sprint and repeated shuttle tests, performed on a sprung wooden floor.

#### Statistical analysis

All statistical analysis was performed using R Studio (v3.5.2). Two-way Analysis of variance was performed for SUM, BEST and DEC, using sex and trial as fixed effects. Cohen’s *d* effect sizes with 95% confidence intervals (CI) were calculated to determine differences between trial 1 and 2 for SUM, BEST and DEC. Coefficients of variation (CV) were determined and two-way mixed effects intraclass correlation coefficients (ICC3) with 95% CI were then performed to assess absolute agreement as recommended by Koo and Li [28]. Here, an ICC of ≥ 0.90 was deemed excellent, >0.75 good, >0.50 moderate and <0.50 poor, with only moderate or better results reported. Where reliability was deemed to be moderate or better, typical error (TE) and standard error of measurement (SEM) were also calculated and compared to smallest worthwhile change (SWC) to determine test sensitivity, where SEM ≤ SWC was determined to be ‘good’ test sensitivity [29, 30]. Bland-Altman plots reporting mean bias and 95% limits of agreement were produced for SUM, BEST and DEC [31]. All reliability analysis was performed separately for female and male data. Pearson’s correlations with 95% CI were conducted to assess the relationship between SUM and other commonly performed physical testing data, including the YoYo Intermittent Recovery Test, 10 m sprint and 40 m sprint time. The Hopkins modified Cohen’s scale was used to describe the relationships [32]. Effects were considered as follows: <0.1 trivial; 0.1–0.3 weak; 0.3–0.5 moderate; 0.5–0.7 strong; 0.7–0.9 very strong; and >0.9 almost perfect. Significance was set at p<0.05.

## Results

**Fig 1:**
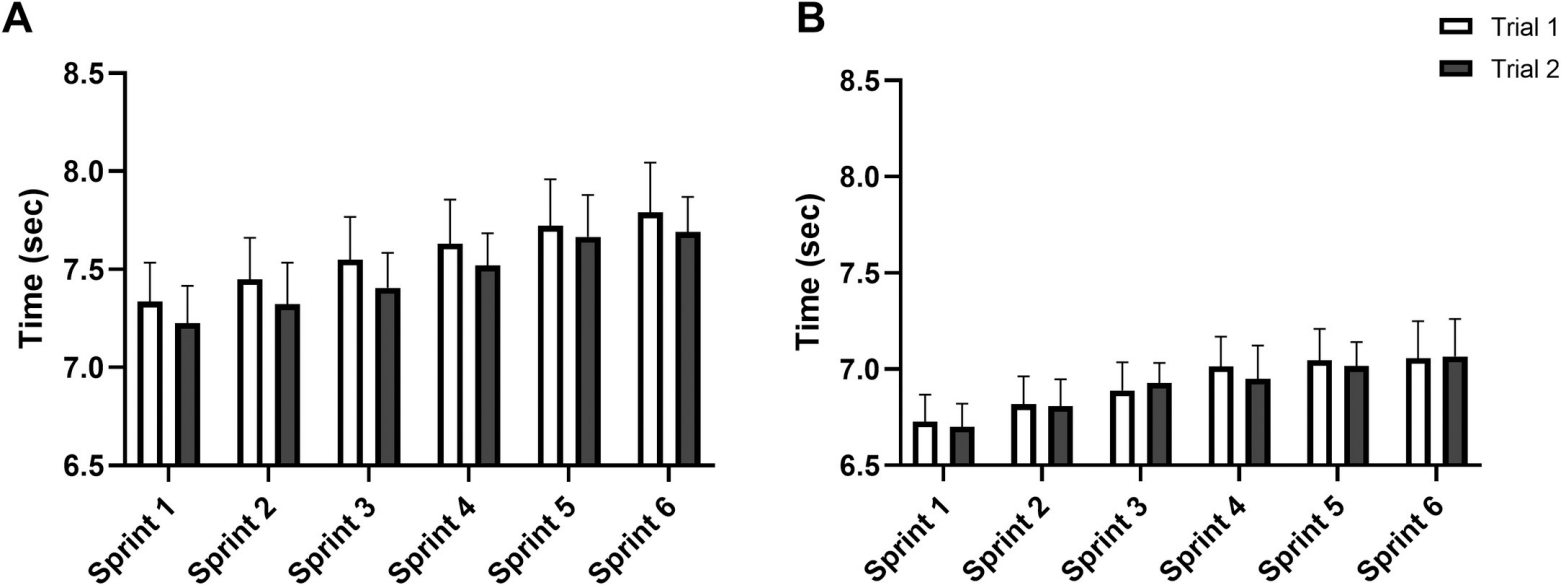
Sprint times during two 6 x 40 m repeated shuttle sprint tests for elite female (A) and male (B) field hockey players.

**Fig 2:**
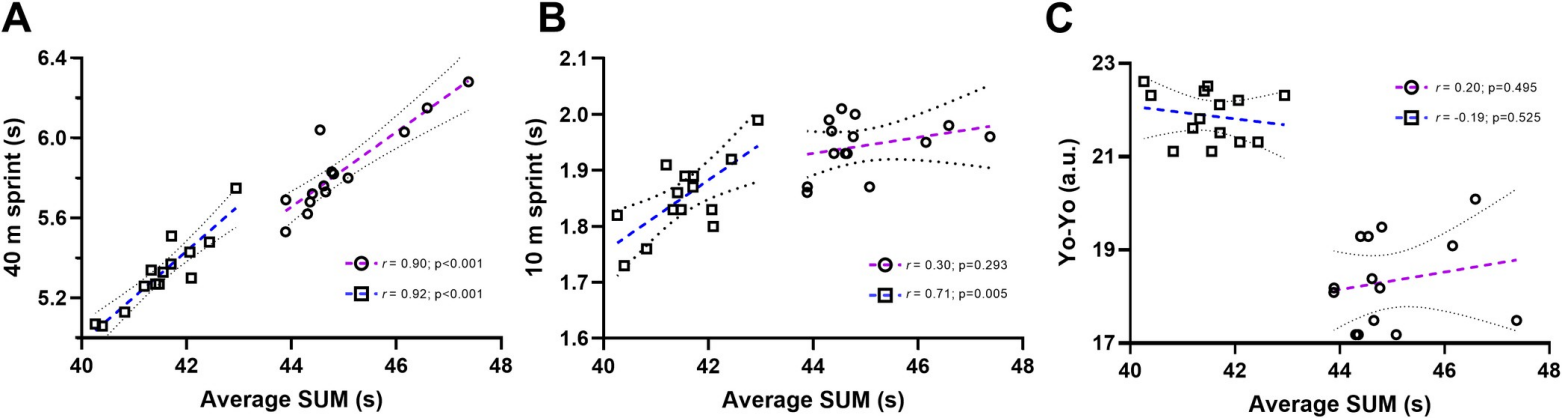
Correlations between the sum of 6 sprints (SUM) during a 6 x 40 m repeated shuttle sprint test and 40 m (A) & 10 m (B) sprint time and YoYo Intermittent Recovery Test Performance (C) for female (circles) and male (squares) elite field hockey players. Purple (female) and blue (male) lines represent the linear regression, with the 95% confidence intervals shown by the black dashed lines.

Summary results for the repeated shuttle sprint test during trials 1 and 2 are presented in Table 1, with sprint-by-sprint data visually represented in Fig 1. In females, ICC scores for both BEST (0.92 [0.80, 0.97]) and SUM (0.94 [0.84, 0.97]) suggests the RSA protocol has ‘excellent’ reliability. TE was 0.05 s and 0.26 s for BEST and SUM, equating to a CV of 0.8% and 0.6%, respectively. No differences in DEC were evident between the two trials (p = 0.337), however a large TE (1.0%) and CV (29%) was apparent. In females, SUM correlated ‘almost perfectly’ with 40 m sprint time (r = 0.90 [0.71, 0.97]; p<0.001, Fig 2), with ‘weak’ relationships evident between SUM and 10 m sprint time (r = 0.30 [-0.27, 0.72]; p = 0.293) and YoYo score (r = 0.20 [-0.37, 0.66]; p = 0.494).

**Table 1:** Reliability results for best sprint (BEST), sum of 6 sprints (SUM) and performance decrement (DEC) in elite female (n = 14) and male (n = 14) field hockey players during a 6 x 40 m repeated shuttle sprint test.

		Trial 1	Trial 2	Difference (95% CI)	*d* ± 95% CI	CV (%)	TE (s)	SEM (s)	SWC (s)	ICC (95% CI)
**BEST**	**Female**	7.29 ± 0.19 s	7.22 ± 0.19 s	-0.06 (-0.02, -0.11)	0.34 ± 0.24	0.76	0.05	0.04	0.04	0.92 (0.80, 0.97)
	**Male**	6.71 ± 0.15 s	6.69 ± 0.12 s	-0.02 (-0.08, 0.03)	0.16 ± 0.40	1.00	0.07	0.03	0.05	0.76 (0.48, 0.90)
**SUM**	**Female**	45.09 ± 1.10 s	44.83 ± 0.99 s	-0.25 (-0.47, -0.03)	0.25 ± 0.21	0.59	0.26	0.20	0.21	0.94 (0.84, 0.97)
	**Male**	41.63 ± 0.79 s	41.43 ± 0.75 s	-0.20 (-0.45, 0.06)	0.26 ± 0.33	0.75	0.31	0.15	0.15	0.84 (0.63, 0.93)
**DEC**	**Female**	3.10 ± 1.23%	3.46 ± 1.11%	0.36 (-0.41, 1.13)	0.31 ± 0.64	29.0	0.95			
	**Male**	3.34 ± 1.46%	3.18 ± 0.84%	-0.17 (-1.31, 0.97)	0.14 ± 0.95	21.5	0.70			

CI = confidence interval, *d* = Cohen’s *d* effect size, ICC = intraclass correlation coefficient, TE = typical error, SEM = standard error of measurement, SWC = smallest worthwhile change, CV = coefficient of variation.

In male players, an ICC of 0.76 [0.48, 0.90] for BEST and 0.84 [0.63,0.93] for SUM indicates the RSA protocol had ‘good’ reliability. Similar TE values were evident for males as seen in females, with 0.07 s and 0.31 s for BEST and SUM respectively. This equates to CVs of 1.0% for BEST and 0.7% for SUM. DEC appeared to be the most inconsistent variable from the RSA protocol, with a high TE (0.7%) and CV (22%). In males, SUM presented a ‘very strong’ positive correlation with 10 m sprint time (r = 0.71 [0.28, 0.90]; p = 0.005, Fig 2) and ‘almost perfect’ correlation with 40 m sprint time (r = 0.92 [0.76, 0.98]; p<0.001, Fig 2). However, only a ‘weak’ negative correlation with YoYo score was apparent (r = -0.19 [-0.65, 0.38]; p = 0.525).

**Fig 3:**
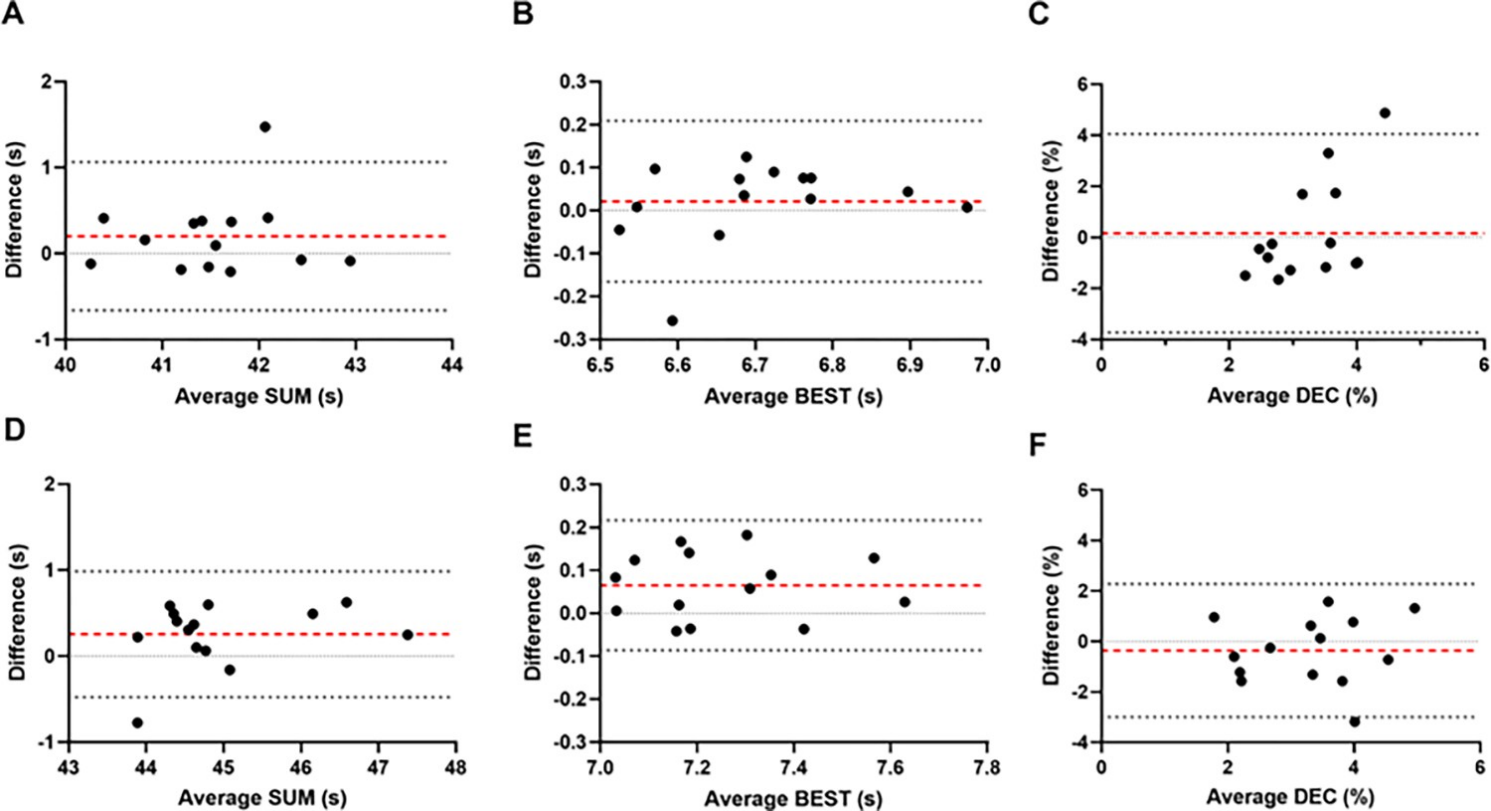
Bland Altman plots calculated for elite male (tiles A-C) and female (tiles D-F) field hockey players for sum of 6 sprints (SUM; tiles A & D), best sprint (BEST; tiles B & E), and performance decrement (DEC; tiles C & F) during a 6 x 40 m repeated shuttle sprint test. Red line represents the bias, with the upper and lower limits of agreement shown by black dashed lines.

There was no sex by trial interaction for BEST (p = 0.625), SUM (p = 0.912) or DEC (p = 0.413). When assessing differences in the RSA protocol between sexes, a main effect for sex in SUM and BEST (both p<0.001) but not DEC (p = 0.939) was evident, indicating that males were able to complete to protocol quicker than females; however, DEC between sexes was similar. There appeared to be minimal bias evident between trials for both males and females across all three variables (Fig 3). The limits of agreement were slightly wider in males (-0.66 to 1.06 s) compared to females (-0.48 to 0.99 s) for SUM (17% wider), with a similar pattern evident for BEST (27% wider), with limits of agreement wider in males (-0.17 to 0.21 s) compared to females (-0.08 to 0.22 s). Finally, DEC showed the largest difference between sexes, with ~47% wider limits of agreement in males (-3.73 to 4.05%) compared to females (-3.00 to 2.28%).

## Discussion

The aim of this investigation was to assess the reliability of a repeated shuttle sprint test in both female and male International field hockey players, and to explore the association of this data with performance in a straight-line, single sprint test and an intermittent, shuttle-based endurance test. The key finding of this investigation was the confirmation of the first hypothesis, that a 6 x 40 m (20 m + 20 m with a 180° turn) repeated shuttle sprint test demonstrates ‘excellent’ (ICC = 0.94) and ‘good’ (ICC = 0.84) reliability when presented as the sum of total sprint time in elite female and male field hockey players, respectively. Additionally, the second hypothesis was confirmed, with ‘almost perfect’ positive correlations found between SUM and 40 m straight line sprint performance in elite female (r = 0.90; p<0.001) and male (r = 0.92; p<0.001) field hockey players, while only ‘weak’ associations were found between repeated shuttle sprint performance and the YoYo Intermittent Recovery Test. Additional findings from this investigation indicate that BEST demonstrates ‘excellent’ (ICC = 0.92) and ‘good’ (ICC = 0.76) reliability in females and males, respectively, while DEC appears unsuitable as a performance measure with elite field hockey players.

While our data suggests DEC should not be used as a performance measure with elite field hockey players, this concurs with previous research reporting DEC during a repeated shuttle sprint test has poor reliability, which is not suitable for evaluating football players [21]. Indeed, the magnitudes of CV for DEC found in females (29.0%) and males (21.5%) in this investigation are similar to previously reported CV (25.0%-36.7%) for repeated shuttle sprint tests [21, 33]. However, both SUM and BEST demonstrated suitable CVs (0.59–1.0%) in females and males, with ‘excellent’ and ‘good’ ICCs reported across sexes. Previous findings in rugby players during a repeated shuttle sprint task have found similar reliability for sum of total sprint times (ICC = 0.95) and best sprint time (ICC = 0.89), with higher CVs (4.54% & 4.47%, respectively) than reported here, which the authors attributed to positional differences in anthropometric characteristics and game demands [23]. Further, research with footballers has found a CV of 1.0% for mean sprint time and 1.6% for best sprint time with a similar repeated shuttle sprint test [21]. Consistent with these earlier works, our data indicate that SUM is the most reliable measure of repeated shuttle sprint performance in elite female and male field hockey players. A typical error of 0.26 s (0.6%) and 0.31 s (0.7%) for females and males, respectively, indicates that a change of approximately 1.0% can be confidently used as a threshold for detection of a real change in repeated shuttle sprint performance [29]. The threshold is slightly higher for BEST in females (TE = 0.05 s; 0.8%), and males (TE = 0.07 s; 1.0%), indicating that a change of ~1.2% and ~1.5%, respectively, is required for a real change to be confidently inferred [29]. This information is vitally important to the interpretation of changes in performance, as small (2.1%) improvements in repeated shuttle sprint performance, close to the threshold required to interpret a real change here, have been reported following a 7-week training intervention [34]. Finally, with SEM ≤ SWC for both SUM and BEST, this test demonstrates ‘good’ sensitivity, indicating the chances of detecting the smallest “important change” are acceptable for use in team sport testing settings [30].

While the assessment of changes in repeated shuttle sprint performance relative to the typical error of the test is important for the appropriate interpretation of the results, it is also important to determine whether the results are providing unique information relative to the rest of the testing battery. In this investigation, it was found that SUM (the most reliable performance measure reported here, and elsewhere) presented an ‘almost perfect’ positive correlation with 40 m straight line running performance in females (r = 0.90; p<0.001) and males (r = 0.92; p<0.001). This association supports similar previous relationships reported between repeated shuttle sprint performance and 30 m straight line sprint performance in football players (r = 0.66) [22], and 40 m straight line sprint performance in rugby players (r = 0.69) [23]. Indeed, performance in a repeated shuttle sprint test has also been reported to correlate with very high-speed running and sprinting distance in professional footballers during match play [20]. This body of work indicates that straight line sprinting speed as a physical quality is highly associated with repeated shuttle sprint performance in a variety of team sports, now including elite-level field hockey, and with high-speed running outputs in football match play. Accordingly, further research in field hockey is now required to determine whether this relationship with match running outputs also exists. Additionally, whether repeated shuttle sprint performance better relates to match running outputs than traditional straight-line repeated sprint tests should also be determined.

In this investigation, 10 m straight line running performance was ‘very strongly’ associated with SUM for males only (r = 0.71; p = 0.005), which supports previous data from male rugby players (r = 0.73) [19]. The ‘weak’ and non-significant correlation between SUM and 10 m straight line running performance (r = 0.30; p = 0.293) in females may indicate that acceleration is less associated with 40 m sprint performance in female populations, or that power-related fitness development is warranted in this population [18], but this requires further investigation. Additionally, the ‘weak’ association between SUM and YoYo Intermittent Recovery Test Level 1 for females (r = 0.20; p = 0.495) and Level 2 for males (r = -0.19; p = 0.525) is contrary to that previously reported in repeated shuttle or repeated straight-line sprint performance for male footballers (r = -0.57 - -0.62; Level 1) [22, 27], field hockey players (r = -0.60; Level 2) [35], and rugby players (r = -0.70; Level 2) [23]. The lack of agreement between our findings with female and male elite field hockey players, and with those of male football, field hockey and rugby players, could be in part explained by the different relative energy system contributions to performance. Brocherie and colleagues have highlighted previously that despite moderate to large associations between YoYo Intermittent Recovery Test Level 2 performance and repeated sprint ability, these tests reflect mechanisms that are likely different [35]. During repeated sprints, there is a greater reliance on muscle phosphocreatine degradation and buffering capacity, and a reduced role of the aerobic energy system compared to the YoYo Intermittent Recovery Test Level 2 [35]. Additionally, differences in the associations between blood morphological and volume indices between repeated sprint and YoYo Intermittent Recovery Test Level 2 performance led the authors to conclude that the tests should not be used interchangeably in a team sport testing battery [35]. Our data supports the use of an intermittent endurance test (such as the YoYo Intermittent Recovery Test) alongside a repeated shuttle sprint test. For females, a straight-line sprinting test with a gate at 10 m to assess acceleration is also warranted to better assess power-related fitness characteristics, while for males, this is associated with performance in a repeated shuttle sprint test, although not as strongly as 40 m sprint times.

A potential limitation of this investigation is that accelerometers were not employed to better describe the acceleration and deceleration characteristics of the test. Additionally, the lack of physiological and perceptual markers associated with these test performances, which may have provided confirmation of maximal effort as well as further insights into the physiological demands imposed. As this was a retrospective analysis of International hockey players performing a regular pre-season physical testing battery, such data was not possible to obtain. Although, the decline in sprint performance from 2^nd^ to 6^th^ sprint found here (1.4±0.1% - 5.6±0.7%) closely agrees with other recent repeated sprint literature in elite field hockey populations (1.0±0.4% - 4.1±0.5%), which supports the fact that maximal efforts were indeed achieved [36]. To avoid detraining, the elite athletes who participated in this investigation were not able to adopt periods of no training leading into both tests; however, the <20 min of exercise performed each day in this investigation represents a marked decrease on regular training loads for these athletes, with the low typical error between trials highlighting the limited role that any fatigue may have played. While an indoor testing location was selected to minimise the impact of the environment on performance, the fact that these tests were not performed on a field hockey pitch in field hockey shoes may have had some impact on change of direction ability which is not able to be confirmed here. Finally, the results of this investigation may not be generalisable to field hockey players of a lower playing level, or team sport athletes in other sports. This highlights the importance of investigations such as these in a range of elite sporting populations, encompassing both female and male athletes.

## Conclusions

In conclusion, the 6 x 40 m repeated shuttle sprint test employed here (20 m + 20 m with a 180° turn) has been demonstrated to be a reliable test which is appropriate to assess International field hockey players. Both best sprint time and sum of 6 sprint times were found to be reliable measures of performance, but the sum of 6 sprint times demonstrated the best reliability, which agrees with previous research on repeated shuttle sprint performance in other team sports. Based on the typical error found here, a 1.0% improvement in sum of 6 sprint times can be confidently interpreted by coaches and practitioners as a real change in performance for both female and male elite field hockey players. Performance in the repeated shuttle sprint test is associated with 40 m straight line sprint performance, but not intermittent endurance performance, and therefore, field hockey testing batteries which include a repeated shuttle sprint test should consider including a test of intermittent endurance.

## Supporting information

S1 Data (XLSX)
